# Associations between Dietary Patterns, *ADR*β*2* Gln27Glu and *ADR*β*3* Trp64Arg with Regard to Serum Triglyceride Levels: J-MICC Study

**DOI:** 10.3390/nu8090545

**Published:** 2016-09-06

**Authors:** Hinako Nanri, Yuichiro Nishida, Kazuyo Nakamura, Keitaro Tanaka, Mariko Naito, Guang Yin, Nobuyuki Hamajima, Naoyuki Takashima, Sadao Suzuki, Yora Nindita, Michiko Kohno, Hirokazu Uemura, Teruhide Koyama, Satoyo Hosono, Haruo Mikami, Michiaki Kubo, Hideo Tanaka, for the Japan Multi-Institutional Collaborative Cohort (J-MICC) Study Group

**Affiliations:** 1 Department of Public Health, Showa University School of Medicine, Tokyo 142-8555, Japan; 2 Department of Preventive Medicine, Faculty of Medicine, Saga University, Saga 849-8501, Japan; ynishida@cc.saga-u.ac.jp (Y.N.); tanakake@cc.saga-u.ac.jp (K.T.); 3 St. Mary’s College Faculty of Nursing, Kurume 830-8558, Japan; k-nakamura@st-mary.ac.jp; 4 Department of Preventive Medicine Nagoya University Graduate School of Medicine, Nagoya 466-8550, Japan; mnaito@med.nagoya-u.ac.jp; 5 Department of Nutritional Sciences, Faculty of Health and Welfare, Seinan Jo Gakuin University, Fukuoka 803-0835, Japan; yinguang@seinan-jo.ac.jp; 6 Department of Healthcare Administration, Nagoya University Graduate School of Medicine, Nagoya 466-8550, Japan; nhamajim@med.nagoya-u.ac.jp; 7 Department of Health Science, Shiga University of Medical Science, Ohtsu 520-2192, Japan; takasima@belle.shiga-med.ac.jp; 8 Department of Public Health, Nagoya City University Graduate School of Medical Sciences, Nagoya 467-8601, Japan; ssuzuki@med.nagoya-cu.ac.jp; 9 Graduate School of Medical and Dental Sciences, Department of International Island and Community Medicine, Kagoshima University, Kagoshima 890-8520, Japan; yora@m.kufm.kagoshima-u.ac.jp; 10 Department of Medicine and Bioregulatory Science, Graduate School of Medical Sciences, Kyushu University, Fukuoka 812-8582, Japan; mkohno@intmed3.med.kyushu-u.ac.jp; 11 Department of Preventive Medicine, Institute of Health Biosciences, the University of Tokushima Graduate School, Tokushima 770-8503, Japan; uemura.hirokazu@tokushima-u.ac.jp; 12 Department of Epidemiology for Community Health and Medicine, Kyoto Prefectural University of Medicine Graduate School of Medical Science, Kyoto 602-8566, Japan; tkoyama@koto.kpu-m.ac.jp; 13 Division of Epidemiology and Prevention, Aichi Cancer Center Research Institute, Nagoya 464-8681, Japan; shosono@aichi-cc.jp (S.H.); tanaka_sec@aichi-cc.jp (H.T.); 14 Division of Cancer Registry, Prevention and Epidemiology, Chiba Cancer Center, Chiba 260-8717, Japan; hmikami@chiba-cc.jp; 15 Center for Integrative Medical Sciences, RIKEN, Yokohama 230-0045, Japan; michiaki.kubo@riken.jp

**Keywords:** dietary pattern, factor analysis, β-adrenergic receptor (*ADR*β), polymorphism, triglyceride

## Abstract

Interactions between dietary patterns and 2 β-adrenergic receptor (*ADR*β) gene polymorphisms (*ADR*β*2* Gln27Glu and *ADR*β*3* Trp64Arg) were examined with regard to the effects on serum triglyceride levels. The cross-sectional study comprised 1720 men and women (aged 35–69 years) enrolled in the Japan Multi-Institutional Collaborative Cohort (J-MICC) Study. Genotyping was conducted using a multiplex polymerase chain reaction-based invader assay. We used 46 items from a validated short food frequency questionnaire and examined major dietary patterns by factor analysis. We identified four dietary patterns: healthy, Western, seafood and bread patterns. There was no significant association between any dietary pattern and serum triglyceride levels. After a separate genotype-based analysis, significant interactions between *ADR*β*3* Trp64Arg genotype and the bread pattern (*p* for interaction = 0.01) were associated with serum triglyceride levels; specifically, after adjusting for confounding factors, Arg allele carriers with the bread pattern had lower serum triglycerides (*p* for trend = 0.01). However, the Trp/Trp homozygous subjects with the bread pattern showed no association with serum triglycerides (*p* for trend = 0.55). Interactions between other dietary patterns and *ADR*β polymorphisms were not significant for serum triglyceride levels. Our findings suggest that *ADR*β*3* polymorphism modifies the effects of the bread pattern on triglyceride levels.

## 1. Introduction

Elevated triglyceride levels have become a major health problem in many countries owing to their causal relationship with chronic disease, such as cardiovascular disease (CVD) and type 2 diabetes mellitus [1,2]. A scientific statement from the American Heart Association indicates that the elevated serum triglyceride is not directly atherogenic but represents an important predictor of CVD risk [3]. The serum triglyceride levels are affected by genetic factors and nutrition as a key environmental factor [3,4]. Polymorphisms of beta-adrenergic receptor (*ADR*β) genes have been ascribed to the control of lipid metabolism and regulation of body fat variability [4]. The glutamine-to-glutamic acid variant at codon 27 of *ADR*β*2* (*ADR*β*2* Gln27Glu) and the tryptophan-to-arginine variant at codon 64 of *ADR*β*3* (*ADR*β*3* Trp64Arg) have been associated with serum triglyceride levels in the Japanese population [5,6,7]. The frequency of Glu allele carriers with the *ADR*β*2* Gln27Glu polymorphism in the Japanese population ranges from 0.05 (5%) to 0.07 (7%) [5,8], which is much lower than those reported in Caucasians [9]. In contrast, the frequency of Arg carriers with the *ADR*β*3* Trp64Arg polymorphism in the Japanese population is about 0.20 [6,7,10], which is lower than that in the Pima Indians [11] but considerably higher than in Caucasians [12,13]. Therefore, the *ADR*β*3* Trp64Arg may play a particularly important role in the regulation of serum triglyceride levels in the Japanese.

Epidemiologic studies on the relationship between diet and disease have traditionally evaluated the effects of single nutrients or foods on disease incidence [14,15]. Recently, dietary pattern analyses, which examine the effects of overall diet, have emerged as an alternative and comprehensive approach for disease-risk analyses [16]. Factor analysis and cluster analysis have been mostly commonly reported as a posteriori approaches to a dietary pattern analysis [16], and another method has used negative matrix factorization to study dietary patterns [17]. Factor analysis is a generic term that includes principal component analysis (PCA) [18]. The PCA is effective at transforming a large number of correlated variables to a smaller number of unrelated variables, whereas factor analysis is concerned with the reduction of a set of observable variables in terms of a small number of latent factors [18]. The factor analysis is a multivariate statistical technique that uses information reported on food frequency questionnaire (FFQ) or in dietary records to identify common underlying dimensions (factors or patterns) of food consumption [16,19,20]. The results of previous studies on the association between dietary patterns and serum triglyceride levels have been inconsistent [21,22,23]. A cross-sectional study showed that a higher score for the Mediterranean diet was associated with lower serum triglyceride levels [23]. Although two Japanese studies have reported an association between dietary patterns and serum triglyceride levels, there was no association between any of three identified dietary patterns (healthy dietary pattern, animal food pattern and Westernized breakfast pattern) and serum triglyceride levels [21,22]. Several previous studies have indicated that polymorphisms of *ADR*β genes modify the effects of nutrients or dietary factors on serum triglyceride levels [24,25,26,27]. However, there has been no study that investigated the combined effects of dietary patterns and these *ADR*β polymorphisms on serum triglyceride levels.

In this study, we examined whether or not there is a joint effect of dietary patterns and *ADR*β*2* Gln27Glu or *ADR*β*3* Trp64Arg on serum triglyceride levels in a Japanese population.

## 2. Materials and Methods

### 2.1. Study Participants and Data Collection

The Japan Multi-Institutional Collaborative Cohort (J-MICC) Study is a large cohort study launched in 2005 to confirm and detect gene-environment interactions in lifestyle-related diseases, mainly cancer. The details of the study procedure have been described elsewhere [28,29]. The subjects of the current study were participants in the J-MICC Study, which was initially conducted in 10 areas of Japan and comprised about 75,000 volunteers aged 35–69 years. For the current cross-sectional study, the data were from 4490 J-MICC Study participants were enrolled in 10 study areas throughout Japan between 2005 and 2008. Of these, we excluded 2770 subjects (1154 men, 1616 women) based on any of the following conditions: (i) lack of genotype data (*ADR*β*2* genotype: 2 men, 3 women; *ADR*β*3* genotype: 3 men, 4 women) or serum triglyceride data (420 men, 659 women); (ii) had taken meals within 8 hours before a blood draw (527 men, 757 women); and (iii) taking cholesterol-lowering medication (75 men, 114 women) or having a history of dyslipidemia (127 men, 79 women). Ultimately, 1720 subjects (955 men, 765 women) remained for the analysis.

Written informed consent was obtained from each participant. The study protocol was approved by the Ethics Committees of Nagoya University School of Medicine and other participating institutions.

Lifestyle factors (smoking status, alcohol consumption, and physical activity) and medical information were evaluated using a self-administered questionnaire. The questionnaire was checked by trained staff to ensure completeness and consistency. For instance, if there was a blank food item on the food frequency questionnaire, the staff asked the participant about the blank item, and they filled in the blank in place of the participant. As another example, if a participant answered “Hypertension” in a question regarding current diseases but left the blank of a following question regarding current medication empty, the staff asked the participant if he/she was currently taking any antihypertensive drugs. Smoking status was classified as current, former, or never. Former smokers were defined as people who had quit smoking for at least 1 year. Alcohol consumption was assessed in 6 of the beverage items and converted into a Japanese sake unit (180 mL), which is equivalent to 23 g ethanol. A validation study of alcohol consumption conducted in the Amami island area (*n* = 66) showed that the Pearson’s correlation coefficients between energy-adjusted alcohol consumption with FFQ and those with 12-day weighed dietary records was 0.71 in men and 0.77 in women [30]. Physical activity was assessed in terms of metabolic equivalents task hours (METs) of daily and leisure-time activity. METs-hours per day (METs·h/day) of daily activity were estimated for heavy physical work and walking. The amount of physical activity was estimated as reported elsewhere [31].

Height and weight were measured to the nearest 0.1 cm and 0.1 kg, respectively. Body mass index (BMI) was calculated as weight (kg) divided by the square of height (m). The serum triglycerides, total cholesterol, high-density lipoprotein cholesterol (HDL-cholesterol) and blood glucose were measured in accordance with standard methods. Low-density lipoprotein cholesterol (LDL-cholesterol) was calculated using the Friedewald formula [32]. Serum triglycerides were measured by the enzymatic method [33] using a Hitachi 7600 Automatic Biochemical Analyzer (Hitachi High-Technologies, Tokyo, Japan).

### 2.2. Dietary Assessment

A validated short FFQ assessed the average intake of 46 foods over the past year, as previously described [34,35,36,37]. Daily amounts of three staple foods (rice, bread and noodles) eaten at breakfast, lunch and supper were estimated using queries about frequencies (six categories: almost none, 1 to 3 times/month, 1 to 2 times/week, 3 to 4 times/week and 5 to 6 times/week, and daily) and amounts per meal (bowls for rice/noodles and slices/rolls for bread). For the other 43 dietary items, only frequency options were given, as follows (assigned daily frequencies in parentheses): almost none (0), 1 to 3 times/month (0.1), 1 to 2 times/week (0.2), 3 to 4 times/week (0.5), 5 to 6 times/week (0.8), 1/day (1), 2/day (2) and ≥3 times/day (3). The intake of total energy (kcal) was calculated using a program developed at the Department of Public Health, Nagoya City University School of Medicine, based on the standard tables of food consumption in Japan (fifth revised edition) [38].

### 2.3. Genotyping

Genotyping was performed as described previously [29]. Single nucleotide polymorphisms (SNPs), including *ADR*β*2* Gln27Glu (rs1042714) and *ADR*β*3* Trp64Arg (rs4994), were genotyped via the multiplex PCR-Invader assay (Third Wave Technologies, Madison, WI, USA) [39] at the Laboratory for Genotyping Development, Center for Genomic Medicine, RIKEN.

### 2.4. Statistical Analysis

All analyses were performed using the SAS statistical software package (Ver. 9.3 for Windows; SAS Institute, Cary, NC, USA). We performed a factor analysis for 46 food items (daily amounts for three staple foods and other foods and beverages in daily frequencies), without adjustment for total energy intake. The four dietary patterns used in the present paper were previously specified in the Japanese population [21,22,40]. The identified factors were rotated by orthogonal transformation (varimax rotation) to improve their interpretability. The factors were selected on the basis of eigenvalues (≥1.0; satisfied for 13 factors) and scree plots (a steep decline in the eigenvalues for the next factor; satisfied for four factors). Ultimately, four factors were identified. The derived factors (dietary patterns) were labeled on based on the food groups, as well as on the relevant literature. The factor scores for each dietary pattern (dietary pattern score) for each individual were estimated as a linear combination of the standardized values for food items and standardized scoring coefficients. Additionally, we performed a sensitivity analysis to examine whether or not the usage of a different method of rotation affected the reproducibility of the present findings. When oblique rotation (promax rotation) was used instead of the abovementioned orthogonal rotation, factor loading showed a similar pattern to that derived from the orthogonal rotation.

The distribution of triglycerides was highly skewed. Therefore, the natural logarithm of triglycerides was used in all analyses, and the resulting geometric means are presented. *ADR*β genotypes were also divided into 2 groups (Gln27Gln or Glu allele carriers for *ADR*β*2* Gln27Glu, and Trp64Trp or Arg allele carriers for *ADR*β*3* Trp64Arg). Because of the considerably low frequency of minor homozygous participants within these groups (*ADR*β*2* Glu/Glu (*n* = 5) and *ADR*β*3* Arg/Arg (*n* = 64)), they were combined with heterozygous participants. To compare the characteristics of participants by genotype, we used the *t*-test for continuous variables and the χ^2^ test for categorical variables. Accordance with Hardy-Weinberg’s equilibrium, which indicates an absence of discrepancy between genotype and allele frequencies, was checked using the χ^2^ test. The adjusted geometric means of triglyceride and the respective 95% confidence intervals (CIs) by tertiles for each dietary pattern score were computed via a general linear model (GLM) in two different multiple linear regression models (Models 1 and 2) to control for potential confounding effects by other factors. A previous study (J-MICC study) reported on the heterogeneity between the study areas. Hara et al. found that the study area had a significant impact on the prevalence of obesity [41]. It is therefore important to consider the heterogeneity between the study areas. We adjusted for study area in the multivariate analyses described here. The first model (Model 1) was adjusted for study area (10 areas), gender (men or women), age (years, continuous), total energy intake (kcal/day, continuous), physical activity (METs·h/day, continuous), alcohol consumption (never, former and current drinker consuming 0.1–22.9, 23.0–45.9 or ≥46.0 g ethanol/day), and smoking (never, former and current smoker of 1–19 or ≥20 cigarettes/day), and the second model (Model 2) was further adjusted for BMI (kg/m^2^, continuous). Since BMI may represent an intermediate step for possible associations between serum triglycerides and dietary patterns, the first and second model were examined separately. The linear trend of association and partial regression coefficients and their 95% CI were assessed by including in the model a continuous variable with the median value of the score within each tertile category, in addition to covariates. The statistical test for interaction was applied to a product term of a dichotomous *ADR*β polymorphisms and each dietary pattern. Ordinal numbers 0 (low level), 1 (middle level) and 2 (high level) were assigned according to the tertile categories of each dietary pattern, and the assigned ordinal numbers were treated as continuous variables for the interaction analyses. A *p* value of < 0.05 was considered to be statistically significant.

## 3. Results

Table 1 shows the basic characteristics of the study subjects by genotype status. The genotype frequencies among the genotyped subjects included in the analyses were in accordance with the Hardy-Weinberg equilibrium for *ADR*β*2* Gln27Glu (χ^2^ = 0.128, *p* = 0.720) and *ADR*β*3* Trp64Arg (χ^2^ = 0.576, *p* = 0.448), and the allele frequencies were similar to those of 4519 subjects [29]. The mean age among *ADR*β*3* Arg allele carriers was significantly higher than that among Trp/Trp homozygotes (*p* = 0.04). The geometric mean of serum triglyceride was significantly lower for *ADR*β*2* Glu allele carriers than for Gln/Gln homozygotes (*p* = 0.04). Other covariates showed no statistically significant differences by genotype.

The results of a factor analysis of the dietary patterns are shown in Table 2. We identified four major dietary patterns: (1) healthy (high in vegetables, fruits other than citrus, *natto* (fermented soybean) and soybean, fish, and bone-edible small fish); (2) Western (high in deep- or stir-fried foods, meat, and mayonnaise); (3) seafood (high in shellfish, fish roe/squid/octopus/shrimp/crab, fish-paste products, and fish); and (4) bread (high in bread and margarine, and low in rice and miso soup). Dietary patterns 1–4 accounted for 12.6%, 7.0%, 5.6%, and 5.1%, of variance in food intakes and together explained 30.3% of the variability.

Table 3 also shows the adjusted geometric means of serum triglyceride and their 95% CIs by tertiles for each dietary pattern score. The healthy pattern was inversely associated with serum triglyceride levels after adjustment for study area, gender, age, total energy intake, physical activity, alcohol consumption status and smoking status (*p* for trend = 0.049); however, additional adjustment for BMI attenuated this association (*p* = 0.08). After adjustment for all factors except BMI, the seafood pattern was positively associated with serum triglyceride levels (*p* = 0.04), but the association was marginal (*p* = 0.11) after further adjustment for BMI. No statistically significant associations between other dietary patterns (Western or bread patterns) and serum triglycerides were observed before or after adjustment for BMI.

We analyzed interactions between dietary patterns and *ADR*β*2* Gln27Glu or *ADR*β*3* Trp64Arg genotypes with regard to effects on serum triglyceride (Table 4). Although there was no interaction between *ADR*β*2* Gln27Glu and the seafood pattern (*p* for interaction = 0.52), this pattern was significantly positively associated with triglyceride levels among Gln/Gln homozygotoes after adjustment for all confounding factors (*p* for trend = 0.03). For *ADR*β*3* Trp64Arg, after adjustment for all factors, there was significant interaction between the bread pattern and *ADR*β*3* Trp64Arg genotype that was associated with serum triglyceride levels (*p* for interaction = 0.01). Inverse associations with serum triglycerides were observed for the bread pattern among Arg allele carriers after adjustment for all confounding variables (*p* for trend = 0.01), but there was no such association for Trp/Trp homozygotes (*p* for trend = 0.55). There were no significant interactions between other dietary patterns and *ADR*β polymorphisms. We also conducted sensitivity analyses in which we compared the pooled effect estimates for subgroups stratified by BMI, alcohol consumption, smoking status, and physical activity. This analysis revealed that the inverse association between the bread pattern and serum triglyceride observed in the Arg allele carriers seems to be clearer in participants with higher physical activity, drinkers, smokers and subjects with obesity (Table 5).

## 4. Discussion

In middle-aged Japanese subjects, we found a significant interaction between the bread dietary pattern (which was characterized by high intake of bread and margarine and low intake of rice and miso soup) and the *ADR*β*3* Trp64Arg SNP on serum triglycerides. In *ADR*β*3* Arg allele carriers, a strong inverse association between the bread pattern and serum triglycerides was observed even after adjustment for covariates, while such an association was not seen among Trp/Trp homozygotes. To our knowledge, there have been no previous reports of this interaction.

The study of dietary patterns, which focuses on a combination of foods, has emerged as a useful way of overcoming the limitations of the single-nutrient/food approach [16]. We identified four dietary patterns (healthy, Western, seafood and bread) by factor analysis. The healthy pattern and Western pattern identified in our study have been consistently observed in previous studies performed in other Japanese populations [21,22,40] and Western populations [42,43]. The seafood pattern identified in this study was similar to that reported by Arisawa et al. [22]. The bread pattern identified in the present study, the opposite of the traditional Japanese staple food pattern (predominantly rice with miso soup and green tea), was similar (Westernized breakfast and bread and daily) to those noted in previous Asian and Japanese studies [21,22,44,45]. Previous studies on the association between dietary pattern and serum triglyceride have been reported [21,22,23]. A cross-sectional study reported an association between a higher score for the Mediterranean diet and lower serum triglycerides [23]. Although two Japanese studies reported an association between dietary patterns and serum triglyceride levels, there were no associations between any of three identified dietary patterns (healthy dietary pattern, animal food pattern, and Westernized breakfast pattern) and serum triglycerides in their Japanese population [21,22]. These results were similar to our own.

In the present study, *ADR*β*3* Arg carriers associated with lower triglyceride levels in the higher bread pattern score, and this association was not seen among *ADR*β*3* Trp/Trp. Although the underlying mechanism is unclear, there may have been some connection with lipid alteration caused by carbohydrate intake. The bread pattern in this study was negatively correlated with carbohydrate intake (*r* = −0.32 in Trp/Trp and *r* = −0.37 in Arg allele carriers), and a previous study made similar observations [45]. However, in previous reports examining the interaction between *ADR*β*3* Trp64Arg and diet and exercise on serum triglyceride, none of the study results showed a statistically significant difference between carriers and noncarriers [24,25,26]. De Luis et al. reported that a three-month reduced carbohydrate intake (carbohydrate % of energy, 38%) affected serum triglyceride levels in both Arg allele carriers and non-carriers; however, there were no marked differences between the effects on serum triglycerides in either carriers or non-carriers [24]. These inconsistencies between previous and present results may be due to differences in ethnic background and/or gender distribution, but we do not have a clear explanation for this.

Frequent intake of coffee represented moderate factor loading on the bread pattern in the present study (Table 2). Greater coffee consumption has been associated with lower serum triglyceride levels in a general Japanese population [46]. Another study in the Japanese showed that the Arg allele of the *ADR*β*3* Trp64Arg polymorphism was associated with lower serum triglyceride levels despite higher visceral obesity [47]. The authors of this previous study speculated that the delivery of lipolytic products from visceral adipose tissue, such as glycerol or non-esterified fatty acid, to the liver, which can increase very-low-density lipoprotein-triglyceride synthesis in the liver, may not be increased but rather decreased in Arg allele carriers [47]. Thus, it is conceivable that both the Arg allele of *ADR*β*3* Trp64Arg and coffee consumption can lead to a reduction in serum triglyceride levels. Based on these previous and present results, we speculate that the coincidence of the Arg allele and coffee consumption might have an additive effect in reducing the serum triglyceride levels, probably through their combined effects to reduce the delivery of non-esterified fatty acid to the liver. Further studies are necessary to understand the precise mechanisms by which the *ADR*β*3* Trp64Arg polymorphism modifies the effects of the bread pattern on serum triglyceride levels.

There was a positive association between the seafood pattern and serum triglycerides among *ADR*β*2* Gln/Gln, but this association was not observed in Glu allele carriers. A Japanese study confirmed an association between seafood pattern and serum triglyceride levels; the seafood pattern scores were not correlated with the prevalence of high serum triglycerides [22]. In the present study, fish had a lower factor loading in the seafood pattern than did other seafood, such as shellfish, fish roe, or squid/octopus/shrimp/crab. In addition, the seafood dietary pattern score was significantly positively correlated with estimated sodium (*r* = 0.28). One cross-sectional study suggests that a higher salt intake might lead to elevated serum triglycerides [48]. Thus, a high intake of salt could lead to higher serum triglycerides, which might partly explain the positive association. The results of this study did not change the association, even after adjustment for sodium intake. However, it is unclear whether or not there is a difference by genotype, as there have been no reports on the different effects of genes on serum triglycerides. More studies are necessary to understand the precise mechanisms underlying the associations observed with the seafood diet.

The strengths of our study include a large sample size and the use of a dietary pattern analysis that better detected the associations of overall diet and genetic predisposition with serum triglyceride. In contrast, some methodological limitations of our study must be discussed. First, due to its cross-sectional design, reverse causation could potentially account for the observed associations; however, we tried to minimize the possibility of reverse causation by excluding participants who were on medication for dyslipidemia. Second, the 4 dietary patterns extracted in the present study explained only 30.3% of the total variance. However, the proportion of variance in the present study was similar to the values reported in previous Japanese studies [21,22,40]. The proportion of variance depends largely on the total number of variables included in an analysis, with total variance being higher in analyses that use fewer variables. Furthermore, our factor analysis was limited in terms of subjectivity in determining and labeling dietary patterns and the difficulty in extrapolating the findings to other populations. Third, covariate variables (e.g. alcohol consumption, smoking status, physical activity and total energy intake) were obtained from a self-reported questionnaire; thus non-differential misclassification may have occurred. Fourth, the current results were based on the subjects who participated in the survey, and there were actually a large number of non-participants; this may have led to selection bias. We analyzed the presence of any differences in the covariates (age, physical activity, alcohol consumption, smoking and BMI) between participants (*n* = 1720) and non-participants (*n* = 2770) and found that all of the above-mentioned covariate factors had significant differences between the participants and non-participants (*p* < 0.05 for all). Finally, although we adjusted for potential confounding factors in the multivariate analysis, residual confounding factors by known or unknown risk factors may have been present.

## 5. Conclusions

The present results suggest that the *ADR*β*3* Trp64Arg polymorphism modifies the effects of the bread dietary pattern on serum triglyceride levels in the Japanese population. Further research is necessary to clarify the mechanisms underlying this association.

## Figures and Tables

**Table 1:** Characteristics of study subjects grouped by genotype status (*n* = 1720).

	*ADR*β*2* Gln27Glu					*ADR*β*3* Trp64Arg				
	Gln/Gln		Glu Allele Carriers		*p*	Trp/Trp		Arg Allele Carriers		*p*
Number (%) ^a^	1525	(88.7)	195	(11.3)		1129	(65.6)	591	(34.4)	
Men	838	(54.9)	117	(60.0)	0.18	640	(56.7)	315	(53.3)	0.30
Women	687	(45.1)	78	(40.0)		489	(43.3)	276	(46.7)	
Age (years) ^b^	54.4	(9.0)	54.2	(8.7)	0.75	54.1	(9.1)	55.0	(8.7)	0.04
Physical activity (METs·h/day) ^c^	9.9	(4.3, 20.5)	8.4	(4.0, 19.5)	0.23	9.6	(4.3, 20.9)	10.0	(4.3, 19.5)	0.70
Alcohol consumption, *n* (%) ^a^										
Nondrinker	597	(40.1)	68	(35.1)	0.58	434	(39.4)	231	(39.9)	0.92
Former drinker	23	(1.6)	2	(1.0)		20	(1.8)	5	(0.9)	
Current drinker										
0.1–22.9 g/day	477	(32.1)	77	(39.7)		361	(32.7)	193	(33.3)	
23.0–45.9 g/day	199	(13.4)	27	(13.9)		153	(13.9)	73	(12.6)	
46.0+ g/day	192	(12.9)	20	(10.3)		135	(12.2)	77	(13.3)	
Smoking, *n* (%) ^a^										
Nonsmoker	867	(56.9)	102	(52.3)	0.20	628	(55.6)	341	(57.7)	0.58
Former smoker	368	(24.1)	53	(27.2)		283	(25.1)	138	(23.4)	
Currnet smoker										
1–19 cigarettes/day	109	(7.2)	9	(4.6)		77	(6.8)	41	(6.9)	
20+ cigarettes/day	181	(11.9)	31	(14.6)		141	(12.5)	71	(12.0)	
Total energy intake (kcal/day) ^b^	1748	(368)	1769	(386)	0.45	1747	(371)	1756	(369)	0.67
Body mass index (kg/m^2^) ^b^	23.2	(3.4)	23.3	(3.3)	0.93	23.1	(3.3)	23.4	(3.5)	0.10
Triglyceride (mg/dL) ^d,e^	94.4	(10.9–619.6)	86.9	(23.0–275.9)	0.04	95.0	(23.0–566.8)	94.3	(11.0–619.6)	0.66
Total cholesterol (mg/dL) ^d^	205.3	(109.0–368.0)	202.7	(132.0–290.0)	0.27	205.0	(109.0–368.0)	205.0	(132.0–318.0)	0.98
HDL-cholesterol (mg/dL) ^d,f^	62.1	(25.9–137.0)	62.0	(34.6–121.3)	0.80	62.3	(25.9–137.0)	61.6	(28.0–121.3)	0.40
LDL-cholesterol (mg/dL) ^d,g^	62.1	(19.3–268.4)	62.0	(19.3–268.4)	0.97	62.3	(19.3–268.4)	61.6	(34.2–216.8)	0.40
Fasting blood glusose (mg/dL) ^d^	97.3	(75.0–301.0)	97.4	(77.0–233.0)	0.78	96.9	(75.0–301.0)	97.9	(34.2–216.8)	0.18
Diabetes, *n* (%) ^a^	41	(2.7)	7	(3.7)	0.70	28	(2.5)	20	(3.4)	0.07
Hypoglycemic medication use, *n* (%) ^a^	36	(2.4)	7	(3.6)	0.30	25	(2.2)	18	(3.1)	0.30

^a^ Number (%); Comparison was based on chi-square test. ^b^ Means (SD); Comparison was based on *t*-test. ^c^ Median (25%, 75%); Comparison was based on Wilcoxon rank-sum test. ^d^ Means (range); Comparison was based on *t* test. ^e^ Triglyceride was log-transformed. ^f^ HDL-cholesterol; high-density lipoprotein cholesterol, ^g^ LDL-cholesterol; low-density lipoprotein cholesterol.

**Table 2:** Factor-loading matrix for the major dietary patterns identified by factor analysis with these study subjects (*n* = 1720).

	Healthy	Western	Seafood	Bread
Rice	-	-	-	**−0.63**
Bread	-	-	-	**0.72**
Noodle	-	-	-	0.29
Margarine	-	-	-	**0.55**
Butter	-	-	-	0.20
Milk	0.35	-	-	-
Yogurt	0.38	-	-	0.25
Miso soup	-	-	-	**−0.53**
Tofu	0.30	-	-	-
Natto and soybean	**0.43**	-	-	-
Egg	-	**0.41**	-	-
Chicken	-	**0.56**	-	-
Beef or pork	-	**0.56**	-	-
Liver	-	-	0.23	-
Ham/sausage/salami/bacon	-	**0.45**	-	0.27
Fish	0.27	-	**0.44**	-
Bone-edible small fish	0.39	-	0.35	−0.23
Canned tuna	-	**0.45**	0.22	-
Squid/octopus/shrimp/crab	-	-	**0.54**	-
Shellfish	-	-	**0.74**	-
Fish roe	-	-	**0.64**	-
Fish-paste products	-	0.24	**0.51**	-
Tofu products	0.29	0.31	0.23	-
Potatoes	**0.55**	0.24	-	-
Pumpkin	**0.49**	-	-	-
Carrots	**0.56**	0.38	-	-
Broccoli	**0.40**	0.20	-	-
Green leafy vegetables	**0.65**	-	-	-
Other green/yellow vegetables	**0.62**	0.26	-	-
Cabbage	**0.54**	0.25	-	-
Daikon (Japanese radish)	**0.58**	-	-	-
Kiliboshi-daikon	0.27	-	0.31	-
Burdock/ bamboo shoot	-	-	0.32	-
Other vegetables	0.39	0.28	-	-
Mushrooms	**0.66**	-	-	-
Seaweed	**0.60**	-	-	-
Mayonnaise	-	**0.53**	-	-
Deep-fried foods	-	**0.61**	-	-
Stir-fried foods	0.21	**0.67**	-	-
Citrus fruit	**0.51**	-	-	-
Other fruit	**0.57**	-	-	-
Peanut	0.25	-	0.21	-
Western-style confectioneries	-	-	-	0.28
Japanese-style confectioneries	0.32	-	-	-
Green tea	0.32	-	-	−0.26
Coffee	-	0.22	-	0.26

For simplicity, factor loadings greater than −0.20 and less than 0.20 are indicated by a dash; those less than or equal to −0.40 or greater than or equal to 0.40 are shown in bold.

**Table 3:** Adjusted geometric means of serum triglyceride by tertiles (Q) of each dietary pattern score in study subjects (*n* = 1720).

Dietary Pattern	Q1 (Lowest)	Q2	Q3 (Highest)	*p* for Trend ^a^	β ^b^
Healthy	572 ^c^	573	575		
Model 1 ^d^	96.3 (92.1–100.5) ^e^	94.0 (90.3–97.9)	90.3 (86.5–94.2)	0.049	−0.037 (−0.074–−0.001)
Model 2 ^f^	95.6 (91.7–99.8)	94.2 (90.6–98.0)	90.6 (87.0–94.4)	0.08	−0.032 (−0.068–0.004)
Western	573	572	575		
Model 1	95.3 (91.4–99.4)	93.6 (89.9–97.5)	91.5 (87.7–95.5)	0.20	−0.023 (−0.059–0.012)
Model 2	95.1 (91.3–99.0)	93.9 (90.4–97.6)	91.4 (87.8–95.2)	0.19	−0.023 (−0.057–0.011)
Seafood	573	572	575		
Model 1	89.0 (85.4–92.7)	96.5 (92.7–100.4)	95.1 (91.3–99.0)	0.04	0.042 (0.001–0.082)
Model 2	89.6 (86.1–93.2)	96.2 (92.6–100.0)	94.7 (91.1–98.5)	0.11	0.032 (−0.004–0.071)
Bread	573	573	574		
Model 1	93.6 (90.2–98.1)	96.3 (92.5–100.2)	90.2 (86.5–94.0)	0.16	−0.020 (−0.054–0.009)
Model 2	93.6 (89.8–97.4)	96.4 (92.7–100.2)	90.5 (87.0–94.3)	0.24	−0.018 (−0.049–0.012)

^a^ Based on multiple linear regression analysis; the model included a continuous variable with the median value of dietary pattern score within each tertile category. ^b^ Partial regression coefficient associated with an increase in 1 category of dietary pattern score (95% confidence interval; 95% CI). ^c^ Number of subjects. ^d^ Adjusted for study area (10 areas), gender (men or women), age (years, continuous), total energy intake (kcal/day, continuous), physical activity (METs·h/day, continuous), alcohol consumption (never, former drinker, or current drinker consuming 0.1–22.9, 23.0–45.9 or ≥46 g ethanol/day), and smoking (never, former smoker, or current smoker consuming 1–19 or ≥20 cigarettes/day). ^e^ Geometric mean (95% CI). ^f^ Adjusted for all variables in Model 1 plus body mass index (kg/m^2^, continuous).

**Table 4:** Interactions between dietary patterns and ADRβ genotypes in relation to serum triglyceride (*n* = 1720).

	***ADR*β*2* Gln27Glu**										***p* for Interaction ^c^**
	**Gln/Gln (*n* = 1525, 88.7%)**					**Glu Allele Carriers (*n* = 195, 11.3%)**					
	**Q1 (Lowest)**	**Q2**	**Q3 (Highest)**	***p* for Trend ^a^**	**β ^b^**	**Q1 (Lowest)**	**Q2**	**Q3 (Highest)**	***p* for Trend**	**β**	
Healthy	506 ^d^	512	507			66	61	68			
Model 1 ^e^	97.4 (92.9–102.1) ^f^	94.8 (90.8–99.0)	91.1 (86.9–95.3)	0.06	−0.039 (−0.078–0.001)	88.2 (77.9–99.8)	87.9 (78.4–98.6)	84.2 (75.1–94.4)	0.58	−0.029 (−0.131–0.073)	0.42
Model 2 ^g^	96.8 (92.5–101.2)	95.1 (91.2–99.1)	91.4 (87.4–95.5)	0.09	−0.034 (−0.072–0.005)	87.5 (77.5–98.7)	88.1 (78.7–98.6)	84.7 (75.7–94.8)	0.67	−0.220 (−0.122–0.079)	0.37
Western	509	503	513			64	69	62			
Model 1	96.6 (92.4–101.1)	94.5 (90.4–98.7)	92.1 (88.1–96.4)	0.17	−0.027 (−0.065–0.011)	86.9 (77.5–97.4)	87.4 (78.5–97.2)	85.8 (76.4–96.3)	0.86	−0.008 (−0.105–0.088)	0.78
Model 2	96.7 (92.6–100.9)	94.7 (90.9–98.8)	91.8 (87.9–95.9)	0.12	−0.029 (−0.066–0.007)	85.6 (76.4–95.8)	88.0 (79.3–97.8)	86.4 (77.1–96.7)	0.95	0.003 (−0.093–0.098)	0.38
Seafood	510	515	500			63	57	75			
Model 1	89.4 (85.5–93.4)	97.1 (93.1–101.4)	96.9 (92.7–101.2)	0.02	0.053 (0.009–0.097)	87.4 (78.0–97.9)	89.7 (79.7–101.0)	83.9 (75.9–92.8)	0.57	−0.032 (−0.143–0.079)	0.69
Model 2	90.0 (86.3–93.8)	96.8 (92.9–100.9)	96.5 (92.5–100.7)	0.03	0.046 (0.003–0.089)	87.6 (78.3–97.8)	90.5 (80.5–101.6)	83.3 (75.2–92.2)	0.47	−0.040 (−0.149–0.069)	0.52
Bread	512	514	490			52	59	84			
Model 1	95.3 (91.2–99.7)	97.4 (93.3–101.7)	90.4 (86.3–94.6)	0.11	−0.028 (−0.062–0.006)	80.9 (71.0–92.1)	89.0 (79.5–99.7)	88.8 (80.5–98.0)	0.35	0.041 (−0.044–0.127)	0.58
Model 2	94.7 (90.8–98.9)	97.4 (93.4–101.5)	91.0 (87.1–95.1)	0.19	−0.022 (−0.055–0.011)	80.9 (71.2–91.9)	89.4 (80.0–99.9)	88.5 (80.4–97.5)	0.37	0.038 (−0.046–0.122)	0.81
	***ADR*β*3* Trp64Arg**										***p* for Interaction**
	**Trp/Trp (*n* = 1129, 65.6%)**					**Arg Allele Carriers (*n* = 591, 34.4%)**					
	**Q1 (Lowest)**	**Q2**	**Q3 (Highest)**	***p* for Trend**	**β**	**Q1 (Lowest)**	**Q2**	**Q3 (Highest)**	***p* for Trend**	**β**	
Healthy	374	384	371			198	189	204			
Model 1	97.5 (92.4–102.9)	93.6 (89.1–98.2)	88.8 (84.3–93.6)	0.02	−0.053 (−0.099–0.007)	95.1 (88.1–102.6)	94.5 (87.7–101.7)	92.2 (85.6–99.3)	0.57	−0.018 (−0.083–0.046)	0.51
Model 2	96.2 (91.3–101.3)	94.0 (89.7–98.6)	89.6 (85.1–94.2)	0.06	−0.042 (−0.086–0.002)	95.7 (88.9–102.9)	94.0 (87.6–101.0)	92.0 (85.7–98.9)	0.49	−0.022 (−0.084–0.040)	0.69
Western	387	373	369			186	199	206			
Model 1	96.4 (91.7–101.3)	93.3 (88.9–98.0)	90.0 (85.5–94.8)	0.07	−0.039 (−0.082–0.004)	93.6 (86.7–101.1)	94.5 (88.0–101.4)	93.5 (86.8–100.7)	0.96	−0.002 (−0.065–0.062)	0.19
Model 2	96.4 (91.9–101.2)	93.1 (88.8–97.6)	90.2 (85.8–94.8)	0.07	−0.038 (−0.079–0.003)	92.9 (86.3–100.0)	95.8 (89.5–102.5)	92.9 (86.5–99.8)	0.93	−0.003 (−0.064–0.059)	0.21
Seafood	353	384	392			220	188	183			
Model 1	88.6 (84.2–93.2)	97.4 (92.9–102.2)	93.6 (89.2–98.2)	0.23	0.030 (−0.019–0.080)	89.4 (83.5–95.8)	94.5 (87.9–101.7)	98.7 (91.6–106.4)	0.06	0.070 (−0.004–0.142)	0.52
Model 2	88.9 (84.6–93.4)	97.1 (92.7–101.8)	93.5 (89.2–97.9)	0.26	0.027 (−0.020–0.075)	90.6 (84.8–96.8)	94.2 (87.8–101.0)	97.6 (90.8–104.9)	0.15	0.052 (−0.018–0.122)	0.59
Bread	372	375	382			201	198	192			
Model 1	91.8 (87.2–96.7)	94.5 (89.9–99.2)	93.5 (88.9–98.3)	0.67	0.008 (−0.030–0.047)	97.7 (90.9–105.1)	99.6 (92.8–107.0)	84.5 (78.4–91.1)	0.01	−0.076 (−0.133–−0.020)	0.01
Model 2	91.5 (87.0–96.2)	94.6 (90.2–99.2)	93.7 (89.2–98.3)	0.55	0.011 (−0.026–0.048)	97.0 (90.4–104.0)	99.8 (93.2–106.9)	85.0 (79.1–91.4)	0.01	−0.069 (−0.123–−0.015)	0.01

^a^ Based on multiple linear regression analysis; the model included a continuous variable with the median value of dietary pattern score within each tertile category. ^b^ Partial regression coefficient associated with an increase in 1 category of dietary pattern score (95% confidence interval; 95% CI). ^c^ Multiplicative interactions between dietary patterns and each genotype. ^d^ Number of subjects. ^e^ Adjusted for study area (10 areas), gender (men or women), age (years, continuous), total energy intake (kcal/day, continuous), physical activity (METs h/day, continuous), alcohol consumption (never, former drinker, or current drinker consuming 0.1–22.9, 23.0–45.9 or ≥46 g ethanol/day), and smoking (never, former smoker, or current smoker consuming 1–19 or ≥20 cigarettes/day). ^f^ Geometric mean (95% CI). ^g^ Adjusted for all variables in Model 1 plus body mass index (kg/m^2^, continuous).

**Table 5:** Interactions between the bread pattern and lifestyle factors by ADRβ3 Trp64Arg genotypes in relation to serum triglyceride (*n* = 1720).

		**Trp/Trp**				***p* for Interaction ^b^**
		**Q1 (Lowest)**	**Q2**	**Q3 (Highest)**	***p* for Trend ^a^**	
Physical activity (METs·h/day)						
	<Median ^c^	163 ^d^	193	200		
		98.4 (91.4–106.0) ^e^	96.3 (90.1–102.8)	94.9 (88.8–101.5)	0.50	0.53
	≥Median	206	178	181		
		85.7 (80.1–91.8)	93.1 (86.8–100.0)	92.5 (86.0–99.4)	0.16	
Alcohol consumption						
	Non drinker	120	141	173		
		81.5 (75.3–88.3)	88.4 (82.4–94.9)	86.3 (80.9–92.1)	0.38	0.88
	Drinker	178	164	154		
		96.7 (89.6–104.3)	98.0 (90.8–105.7)	97.9 (90.3–106.2)	0.83	
Smoking						
	Non smoker	311	283	317		
		87.2 (82.7–91.9)	90.2 (85.5–95.1)	88.5 (84.0–93.1)	0.75	0.92
	Smoker	61	92	65		
		113.2 (98.0–130.6)	115.3 (103.3–128.6)	117.1 (102.0–134.5)	0.75	
BMI (kg/m^2^)						0.71
	<25.0	257	292	292		
		85.4 (80.3–90.8)	89.4 (84.6–94.4)	88.5 (83.6–93.7)	0.46	
	≥25.0	115	82	90		
		112.4 (102.8–122.9)	111.5 (100.5–1236)	108.5 (98.1–120.1)	0.63	
		**Arg Allele Carriers**				***p* for Interaction**
		**Q1 (Lowest)**	**Q2**	**Q3 (Highest)**	***p* for Trend**	
Physical activity (METs·h/day)						
	<Median	91	91	105		
		94.1 (84.6–104.6)	110.7 (99.7–122.9)	91.3 (82.6–100.9)	0.58	0.15
	≥Median	109	106	87		
		96.6 (88.5–105.6)	90.6 (83.1–98.7)	78.9 (71.5–87.0)	<0.05	
Alcohol consumption						
	Non drinker	75	75	81		
		82.6 (74.0–92.2)	95.7 (86.0–106.4)	93.5 (88.9–98.3)	0.70	0.19
	Drinker	103	84	83		
		102.4 (92.8–112.9)	102.1 (92.1–113.3)	82.6 (74.0–92.2)	<0.05	
Smoking						
	Non smoker	160	165	154		
		90.5 (83.8–97.8)	92.5 (85.9–99.6)	82.7 (76.3–89.6)	0.12	0.23
	Smoker	41	33	38		
		139.9 (118.0–165.8)	129.0 (108.3–153.6)	98.1 (83.1–115.7)	<0.05	
BMI (kg/m^2^)						
	<25.0	142	148	145		
		88.0 (80.8–95.8)	93.5 (86.3–101.3)	82.0 (75.3–89.2)	0.23	0.15
	≥25.0	59	50	47		
		126.9 (110.6–145.5)	119.5 (103.4–138.1)	92.8 (79.4–108.5)	<0.05	

^a^ Based on multiple linear regression analysis; the model included a continuous variable with the median value of dietary pattern score within each tertile category. ^b^ Multiplicative interactions between dietary patterns and each lifestyle factors. ^c^ Median value. ^d^ Number of subjects. ^e^ Geometric mean (95% confidence interval). Adjusted for study area (10 areas), gender (men or women), age (years, continuous), body mass index (kg/m^2^, continuous), total energy intake (kcal/day, continuous), physical activity (METs·h/day, continuous), alcohol consumption (never, former drinker, or current drinker consuming 0.1–22.9, 23.0–45.9 or ≥46 g ethanol/day), and smoking (never, former smoker, or current smoker consuming 1–19 or ≥20 cigarettes/day).
